# The Gut Microbiota and Colorectal Cancer: Understanding the Link and Exploring Therapeutic Interventions

**DOI:** 10.3390/biology14030251

**Published:** 2025-02-28

**Authors:** Imen Zalila-Kolsi, Dhoha Dhieb, Hussam A. Osman, Hadjer Mekideche

**Affiliations:** 1 Faculty of Medical and Health Sciences, Liwa College, Abu Dhabi P.O. Box 41009, United Arab Emirates; hussam.balloula@lc.ac.ae (H.A.O.); hadjerme98@gmail.com (H.M.); 2 College of Pharmacy, QU Health, Qatar University, Doha P.O. Box 2713, Qatar; dhoha.dhieb@qu.edu.qa

**Keywords:** colorectal cancer, gut microbiota, pathophysiology, diagnostic strategies, probiotics

## Abstract

CRC remains a significant public health challenge due to its high prevalence and mortality rates. Emerging evidence highlights the critical role of the gut microbiota in both the pathogenesis of CRC and the efficacy of treatment strategies, including chemotherapy and immunotherapy. Dysbiosis, characterized by imbalances in microbial communities, has been implicated in CRC progression and therapeutic outcomes. This review examines the intricate relationship between gut microbiota composition and CRC, emphasizing the potential for microbial profiles to serve as biomarkers for early detection and prognosis. Various interventions, such as prebiotics, probiotics, postbiotics, fecal microbiota transplantation, and dietary modifications, aim to restore microbiota balance and shift dysbiosis toward eubiosis, thereby improving health outcomes. Additionally, the integration of microbial profiling into clinical practice could enhance diagnostic capabilities and personalize treatment strategies, advancing the field of oncology. The study of intratumoral microbiota offers new diagnostic and prognostic tools that, combined with artificial intelligence algorithms, could predict treatment responses and assess the risk of adverse effects. Given the growing understanding of the gut microbiome–cancer axis, developing microbiota-oriented strategies for CRC prevention and treatment holds promise for improving patient care and clinical outcomes.

## Simple Summary

Colorectal cancer (CRC), a leading global cause of mortality, is strongly influenced by gut microbiota, which affects disease progression and treatment response. Our research investigates the impact of gut dysbiosis on the efficacy of chemotherapy and immunotherapy, suggesting the utility of microbial profiling for early CRC detection and prognosis. We explore interventions including probiotics, prebiotics, and dietary changes to rebalance gut microbiota and enable personalized treatment strategies. Furthermore, we assess the integration of intratumoral microbiota analysis with artificial intelligence to enhance treatment precision, aiming to improve patient outcomes and reduce CRC incidence and severity through personalized care approaches.

## 1. Introduction

Colorectal cancer (CRC), which includes both colon and rectal cancers, ranks as the third most lethal and diagnosed cancer globally, accounting for about 9.4% of cancer-related deaths in 2020 [1]. In 2019, cancer was reported to cause 250 million Disability Adjusted Life Years (DALYs), approximately 10 million deaths, and 23.6 million new diagnoses worldwide, reflecting significant increases since 2010 [2]. Projections by the International Agency for Research on Cancer (IARC) anticipate a 56% rise in CRC cases and a 69% increase in fatalities by 2040 [3]. CRC research, particularly concerning microbiomes and cancer, is vital. The disease’s progression, influenced by genetic and multifactorial factors, is linked to microorganisms, including the gut microbiota, which harbors nearly 100 trillion microbes and produces around 200 times more genes than the human genome [4]. This diverse “organ” is crucial for maintaining homeostasis by aiding digestion, producing metabolic byproducts, and supporting immune tolerance [4]. Short-chain fatty acids (SCFAs) produced by gut bacteria play a pivotal role in health, with deficiencies linked to CRC and other conditions [5]. The fermentation of dietary fibers produces short-chain fatty acids (SCFAs), such as butyrate, which have anti-inflammatory and anti-carcinogenic qualities by blocking histone deacetylases (HDACs), which activate tumor suppressor genes [6]. On the other hand, by producing reactive oxygen species (ROS) and triggering pro-inflammatory pathways, secondary bile acids such as deoxycholic acid (DCA) can cause DNA damage and encourage carcinogenesis [7].

Confounding elements such as diet, antibiotic consumption, and geographical differences greatly influence microbiota research. Diet affects the composition and function of microbes, whereas antibiotics may lead to enduring changes in the gut microbiome [8]. Geographical differences influence microbiota diversity owing to variations in lifestyle, environment, and genetic factors. To address these confounders, we suggest implementing standard operating procedures that involve uniform dietary controls, comprehensive records of antibiotic usage, and stratification by geographic area in study designs [9]. These actions will improve the consistency and dependability of microbiota studies.

**Figure 1:**
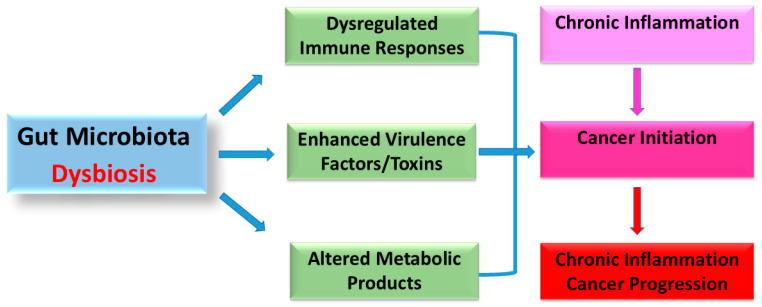
Dysbiosis of the gut microbiota and its connection to CRC. Three mechanisms, comprising the dysregulation of immune responses, virulence factors/toxins, and metabolic products, could be used to induce chronic inflammation and, consequently, the beginning and progression of cancer via dysbiosis of the gut microbiota and an increase in the quantity of pathogenic bacteria.

**Figure 2:**
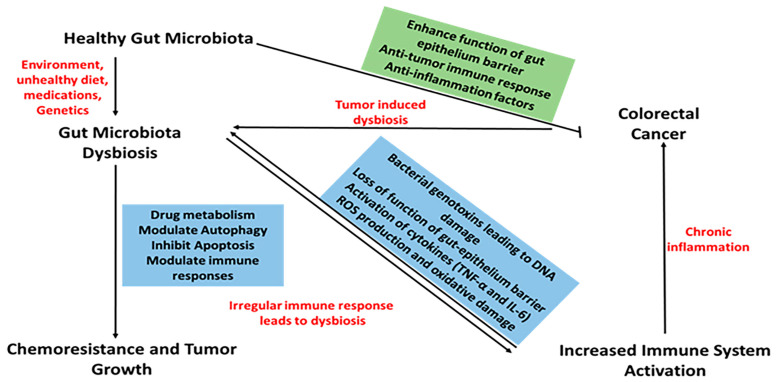
Relationship between gut microbiota dysbiosis, colorectal cancer, immune system, and chemoresistance. Numerous factors impact dysbiosis of the gut microbiota, which in turn affects a number of processes, including immune system activation, cancer progression, and the emergence of chemoresistance.

Gut microbiota synthesizes substances with anti-inflammatory and antioxidant properties but can also produce harmful compounds, like carcinogens [3]. Dysbiosis, or microbiota imbalance, can provoke inflammation and cancer through immune dysregulation and metabolic product influence, contributing to CRC pathogenesis [10,11] (Figure 1). Disrupted microbiota composition relates to CRC through genomic and epigenomic alterations, immune response induction, and inflammatory regulation [12] (Figure 2). Understanding this relationship enhances CRC management, with gut microbiota serving as potential biomarkers for screening and treatment efficacy modifiers [13,14]. For instance, “*Fusobacterium nucleatum*” is prevalent in CRC patients, suggesting its use in early detection [3]. Recent research has shown particular microbial signatures linked to CRC, such as the enrichment of *Parvimonas micra* and *Fusobacterium nucleatum*, which are consistently seen in several cohorts [15]. In comparison to traditional biomarkers such as the fecal immunochemical test (FIT), these microbial indicators have demonstrated promise in the non-invasive diagnosis of CRC, with some studies claiming improved sensitivity and specificity.

This report explores the link between CRC and gut microbiota, focusing on etiology, pathophysiology, and biological mechanisms. It also examines recent advances in pathogenetic understanding and potential therapeutic interventions targeting the gut microbiome and associated metabolic pathways.

## 2. Colorectal Cancer Pathophysiology

Colorectal cancer (CRC) is a highly heterogeneous disease with distinct genetic and molecular profiles across its subtypes [16]. The development and progression of CRC are driven by genetic events categorized into three primary molecular pathways: chromosomal instability, microsatellite instability, and CpG Island Methylator Phenotypes [17]. Key molecular drivers include mutations and gene amplifications in *KRAS*, *BRAF* V600E, and *HER2* [18]. In 2015, the Colorectal Cancer Consortium introduced a classification system based on clinical and multiomics data, identifying four Consensus Molecular Subtypes (CMSs) considered the most accurate:

“CMS1”, associated with microsatellite instability and immune-related gene upregulation.

“CMS2”, characterized by RAS protein mutations.

“CMS3”, which displays metabolic dysregulation, with heightened lipid synthesis and glutaminolysis, alongside KRAS overexpression mutations.

“CMS4”, linked to epithelial–mesenchymal transition [18].

Approximately 80–85% of CRC cases are sporadic, not arising from hereditary mutations but likely influenced by environmental factors such as lifestyle, diet, and specific microorganisms [16]. Sporadic CRC often begins with mutations in the adenomatous polyposis coli (*APC*) gene, triggering a cascade leading to cancer development [16]. Notably, around 20% of cancers are linked to pathogens like “*Helicobacter pylori*”, hepatitis B and C viruses, and human papillomavirus [19]. Significant microbiota composition differences exist between healthy individuals and those with cancer, highlighting the role of microorganisms in CRC [20]. Research has indicated that the gut microbiota of patients with colorectal cancer is less diverse than that of healthy people. The development of CRC is linked to this decline in microbial diversity [21]. Patients with colorectal cancer have notable changes in specific bacterial populations. For instance, CRC patients have much-reduced levels of good bacteria like *Bifidobacterium* and higher levels of potentially dangerous bacteria like *Fusobacterium nucleatum.* Furthermore, healthy people and CRC patients have different gut microbiota functional capacities. Pro-inflammatory and carcinogenic chemicals are produced more frequently in CRC patients, which may aid in the growth and spread of tumors [21,22].

## 3. Impact of the Gut Microbiome on the Genome and Epigenome of Colon Epithelial Cells

**Figure 3:**
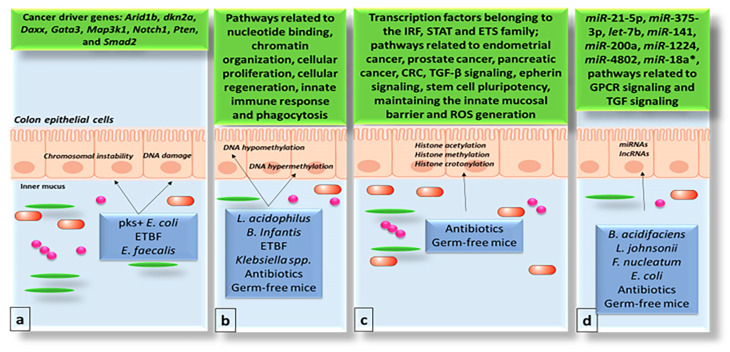
The gut microbiome’s impact on colon epithelial cells’ (CECs) genome and epigenome. (**a**). Specific microbes cause DNA damage in CECs through their toxins. Other microbes affect macrophages, leading to chromosomal instability and mutations in cancer-related genes. (**b**). Gut microbes, shown using antibiotics and germ-free mice, trigger gene hypermethylation and hypomethylation in CRC pathways. (**c**). Gut microbes, demonstrated with antibiotics and germ-free mice, do not typically affect global chromatin structure in CECs but alter promoter and enhancer regions in CRC pathways. (**d**). Gut microbes, shown using antibiotics and germ-free mice, influence the expression of oncomiRNAs and anti-oncomiRNAs in CECs. Abbreviations: pks (Polyketide Synthase), ETBF (Enterotoxigenic *Bacteroides Fragilis*), IRF (Interferon Regulatory Factor), STAT (Signal Transducer and Activator of Transcription), ETS (E26 Transformation-Specific), CRC (Colorectal Cancer), TGF (Transforming Growth Factor), TGF-β (Transforming Growth Factor Beta), and GPCR (G Protein-Coupled Receptor). Symbols: The “*” indicates the passenger strand of the miRNA duplex. A circle symbol represents spherical cocci bacteria, while an ellipse symbol represents rod-shaped bacilli bacteria.

Emerging research highlights the significant role of gut microbiota in colorectal cancer (CRC). Microbiota-related biomarkers are gaining traction as non-invasive tools for CRC prevention, diagnosis, and treatment [23,24,25,26]. Reviews and meta-analyses indicate that specific bacterial species and gut imbalances are linked to CRC development, as observed in patients and animal models [24]. Gut dysbiosis escalates pathogenic microbial metabolic activity, leading to bile acid deconjugation and increased secondary bile acids like deoxycholic acid, which are carcinogenic [23]. Procarcinogenic metabolites, such as sulfides, ammonia, phenols, and nitrosamines, produced during bacterial fermentation and dietary sulfate reduction, may promote CRC. Metabolic byproducts of gut microbes can incite inflammatory responses and produce reactive oxygen species and toxins, including tumor necrosis factor-alpha, interleukin-6, and various cytokines. These products can damage DNA and impair epithelial cell function [26]. Figure 3 illustrates the gut microbiome’s influence on the genome and epigenome of colon epithelial cells.

## 4. The Gut Microbiome and Chromatin Structure

In non-dividing eukaryotic cells, DNA wraps around histones to form nucleosomes, a key component of chromatin. Histones are composed of core proteins H2A, H2B, H3, and H4. Post-translational modifications, such as the acetylation and methylation of histone tails, are crucial for gene expression regulation [27]. In cancer, mutations in histone modifiers can disrupt these epigenetic marks. Histone acetylation, controlled by histone acetyltransferases and deacetylases (HDACs), modifies chromatin structure by reducing DNA–histone interactions. Hyper-acetylation can activate oncogenes, while hypo-acetylation can silence tumor suppressor genes [28]. The gut microbiome’s impact on chromatin modification in colorectal cancer (CRC) is intricate. Butyrate-producing bacteria have shown protective effects in CRC mouse models by inducing chromatin changes that affect gene expression. Some microbes can specifically influence local chromatin, rather than global accessibility. For example, histone crotonylation, linked to gene expression in cancers including CRC, decreases post-antibiotic treatment, correlating with reduced short-chain fatty acids and lower *HDAC2* expression [29,30]. In AOM/DSS-induced CRC models, researchers found that germ-free mice given the butyrate-producing bacterium “*Butyrivibrio fibrisolvens*” along with a high-fiber diet showed significant tumor protection. Mice receiving only the bacterium or the diet showed less protection, whereas those with a mutant, low-butyrate strain had intermediate results. These changes increased transcription factors binding site accessibility, correlating with CRC progression [31]. Butyrate, a short-chain fatty acid generated from the fermentation of dietary fiber in the intestines, has various mechanisms that aid in tumor protection. Butyrate boosts the immune system’s capacity to identify and eliminate cancer cells by stimulating the function of regulatory T cells and lowering inflammation. It promotes an anti-inflammatory setting in the intestines, potentially preventing the proliferation and dissemination of cancer cells. Moreover, butyrate can trigger apoptosis (programmed cell death) in cancer cells, thus decreasing tumor growth. Moreover, by blocking HDACs, butyrate can modify gene expression in cancer cells, resulting in decreased proliferation and heightened cell death. Butyrate enhances the intestinal barrier, stopping harmful substances from entering the bloodstream and possibly leading to inflammation or cancer. These mechanisms together play a role in butyrate’s ability to safeguard against tumor formation and advancement [32].

## 5. The Gut Microbiome and Non-Coding RNAs

Approximately 98% of the genome’s non-protein-coding portion regulates gene expression, impacting physiological development and disease progression [33,34,35,36,37,38]. Non-coding RNAs (ncRNAs) are key transcriptional regulators that can be spliced post-transcriptionally but do not encode proteins. Their roles vary with tissue context, exhibiting pro- or anti-tumorigenic effects [28]. NcRNAs are generally divided into small ncRNAs (snRNAs), such as microRNAs (miRNAs), and long ncRNAs (lncRNAs). miRNAs, usually 18–25 nucleotides long, are highly researched in colorectal cancer (CRC). They can undergo genetic and epigenetic modifications that alter their expression, distinguishing between cancerous and normal tissues [28,29]. During CRC progression, miRNAs may be upregulated or downregulated, often acting as onco-miRs by inhibiting tumor suppressors or as ts-miRs by suppressing oncogenes [29]. LncRNAs, such as *HOTAIR*, *CCAT*, *MALAT-1*, and *H19*, are involved in CRC-related processes like stem cell renewal, tumor infiltration, and metastasis, and are significant for diagnosis and prognosis [39]. They engage in various cancer pathways, including *WNT*, *EGFR*, *TGF-β*, and *p53*, influencing CRC development [28]. Gut microbiota studies using germ-free and conventional mice have shown a role in regulating miRNA expression in colorectal epithelial cells. NanoString and qRT-PCR analyses link gut microbes to reduced fecal miRNA expression, with conventional mice showing higher levels of certain miRNAs compared to germ-free mice [29,40]. Antibiotic-treated rats displayed temporal miRNA expression changes, yet their functional implications remain uncertain [41]. *miR-21-5p*, an oncomiRNA, is elevated in the intestines of conventional mice and further increases upon bacterial stimulation. In contrast, *miR-375-3p*, crucial in intestinal stem cells, is downregulated in CRC, and its suppression promotes cell proliferation [42]. Studies have suggested miRNA and microbe interactions in CRC, with “*Fusobacterium nucleatum*” affecting chemotherapy resistance and tumor progression [43]. While gut microbe-induced miRNA modifications during CRC are documented, the practical consequences on tumor development need further exploration.

## 6. Recent and Future Diagnostic Strategies

### 6.1. Urine-Derived Extracellular Vesicles (EVs)

Yoon et al. utilized next-generation sequencing to analyze urine samples for bacterial-derived extracellular vesicles (EVs) from a cohort of 207 individuals, including both control and colorectal cancer (CRC) models [44]. EVs are produced by bacteria to facilitate communication with host cells and other bacterial species, and they are commonly excreted in urine after circulating through the vascular system and colonic mucosa. These EVs are considered an indirect measure of bacterial activity as they contain bacterial DNA, providing insights into microbial interactions. Recent studies have suggested that bacterial metabolites and activity may hold greater significance than bacterial composition in the pathogenesis of CRC. This study focused on evaluating EVs from the urine of CRC patients to assess their potential as biomarkers for early CRC diagnosis. Findings indicated an increase in specific bacterial species known to be implicated in CRC pathogenesis, such as “*Staphylococcus*” species and “*Acinetobacter*”, alongside a decrease in “*Akkermansia muciniphila*” and “*Bacteroides*”, which are known to have protective roles against colorectal cancer. *Staphylococcus* can generate toxins that induce inflammation and cause DNA damage, thereby aiding in CRC [45]. *Acinetobacter* is recognized for its involvement in antibiotic resistance and infections, potentially leading to inflammation and impairing the gut barrier [46]. *Akkermansia muciniphila* typically offers protection, preserving gut barrier integrity and influencing the immune response. Species of *Bacteroides*, including *Bacteroides fragilis*, can generate harmful toxins, whereas some produce helpful substances like butyrate [47]. These interactions can either be damaging or protective, based on the circumstances.

Furthermore, utilizing urine-derived EVs presents a more convenient collection method for patients compared to other body fluid collection procedures, highlighting the potential for this approach to serve as an early diagnostic tool for CRC [44].

### 6.2. Histopathology Imaging Using Artificial Intelligence

Artificial intelligence (AI) has revolutionized colorectal cancer (CRC) diagnosis with the development of weakly labeled supervised deep learning methods using the Inception-v3 convolutional neural network (CNN). This approach enhances diagnostic accuracy and efficiency, leveraging data from sources like The Cancer Genome Atlas (TCGA) and the National Centre for Tumor Diseases (NCT) to train models that learn histological morphology for accurate cancer detection [48]. A human–AI trial showed AI equaling pathologist diagnoses in speed and accuracy, using FFPE samples to generalize results across CRC and possibly other cancers. The AI also incorporated a heatmap to highlight potential cancer areas on whole-slide images, diagnosing cases in about 13 s with datasets from diverse locations ensuring robustness [48]. In parallel, AI is being investigated to predict microsatellite instability (MSI) and mismatch repair deficiency (dMMR) directly from tissue slides, crucial for identifying Lynch Syndrome and guiding immunotherapy treatment. Hildebrand et al. explored AI’s ability to classify dMMR/MSI, evaluating its accuracy and impact on treatment predictions [49]. Takamatsu et al. developed a machine learning model for assessing lymph node metastasis (LNM) in T1 CRC cases, aiming to mitigate interobserver variability [50]. Using CNNs for plaque extraction and Random Forest algorithms for LNM prediction, this model evaluated 783 T1 CRC cases, achieving predictive insights [50]. Additionally, Shimada et al. utilized CNNs on H&E slides for tumor mutational burden (TMB) prediction [51], with deep learning also applied to identify tumor biomarkers, mutant genes, and metastasis status from histopathological images [52,53,54]. While feasible, integrating H&E data with clinical information for enhanced TMB prediction remains a potential future research direction to improve CRC diagnostics and treatment planning. Recent developments in artificial intelligence (AI) have greatly improved microbiome-focused diagnosis and the customization of therapy. AI models like DeepMicro leverage high-throughput sequencing data to identify patterns, classify microorganisms, and forecast disease associations [55]. For example, Meta-Spec combines host and microbial data to illustrate the relationship between particular microbiome patterns and specific diseases [56]. Moreover, machine learning methods such as Random Forest and Support Vector Machines have been utilized to detect microbial signatures linked to different health conditions, enhancing diagnostic precision [57]. These AI-based methods have shown greater sensitivity and specificity than traditional biomarkers, establishing them as important resources in personalized medicine.

### 6.3. Engineered Bacteria to Detect Cancer in the Gut

Traditional non-invasive colorectal cancer (CRC) tests, like fecal immunochemical testing, often lack precision, while colonoscopies, though accurate, are expensive and risky, limiting widespread screening to those over 45. A pioneering study used CRISPR technology to engineer “*Acinetobacter baylyi*”, a benign soil bacterium, into a biosensor capable of detecting CRC-associated DNA mutations in the gut. This innovative method, known as “CATCH”, demonstrates the potential for engineered bacteria to non-invasively identify tumor DNA, advancing CRC screening [58,59]. The CATCH technique utilizes the natural competence of “*A. baylyi*” to incorporate tumor DNA into its genome, effectively acting as a biosensor for disease biomarkers in bodily fluids. Engineered bacteria have been shown to accurately identify cancer mutations, marking a significant step in early cancer detection [58,59]. An advanced biosensor has also been developed to detect mutations in non-engineered DNA, employing a transcriptional repressor and reporter construct for specificity [60]. While clinical application requires further optimization, this promising approach paves the way for non-invasive early-stage cancer diagnostics and enhanced treatment strategies. The method involves administering modified “*A. baylyi*” rectally to mice with CRC, where the bacteria uptake tumor DNA shed by cancer cells. Antibiotic selection processes facilitate the identification of bacteria that integrate tumor DNA, rendering them kanamycin-resistant. Although the approach is adaptable for various cancers, challenges like public acceptance and safety persist. Future efforts will focus on enhancing method efficiency, improving biosensors, exploring oral delivery, and ensuring safety, aiming to provide non-invasive diagnostics and personalized treatment options [60].

## 7. Treatment Implications

To minimize toxicity to normal tissues, prevention and treatment strategies for colorectal cancer (CRC) targeting metabolic processes require a well-defined therapeutic index. CRC treatments vary by stage, from surgical resection in stage I to chemotherapy, often with 5-Fluorouracil (5-FU), in advanced stages. While 5-FU inhibits DNA replication, new therapies are exploring metabolic vulnerabilities, particularly in *KRAS* mutant CRC. Promising options include the dual *RAS/MEK* inhibitor RO5126766 and the combination of l-asparaginase with rapamycin to inhibit asparagine synthetase. Additionally, vitamin C has been shown to induce oxidative stress in glycolytic *KRAS* and *BRAF* mutant CRC cells. Targeting glycolysis with 2-deoxy-d-glucose (2-DG) and the antidiabetic drug metformin has also proven effective against CRC [61]. Future treatment strategies may focus on identifying metabolic weaknesses in immune cells, tailoring therapies to specific genetic alterations, and addressing metabolic changes during cancer progression. The administration of specific metabolites, like β-hydroxybutyrate, could further enhance treatment outcomes [61]. These approaches aim to deliver personalized, effective interventions that improve patient outcomes while minimizing side effects.

## 8. Potential Applications of the Gut Microbiome in CRC Prevention and Treatment

A healthy gut microbiome is marked by beneficial species that degrade complex polysaccharides into lactic acid and other compounds, crucial for maintaining intestinal equilibrium. In colorectal cancer (CRC), dysbiosis manifests as a shift towards CRC-associated bacteria and a reduction in probiotic lactic acid bacteria (LAB) like “*Bifidobacterium*”, “*Lactobacillus*”, and “*Streptococcus*” [16]. Modifying the gut microbiota composition in those at high risk for CRC may aid in disease prevention and enhance chemotherapy and immunotherapy efficacy. Strategies such as probiotics, prebiotics, postbiotics, fecal microbiota transplantation (FMT), and bacteriophage therapy aim to restore bacterial balance and improve metabolic functions, fostering a healthier gut environment [3]. These biotherapeutic approaches not only contribute to CRC prevention but also optimize therapeutic responses and reduce the side effects of conventional treatments, thereby improving patient outcomes.

### 8.1. Probiotics

Probiotics, which include bacteria, yeasts, and molds, confer health benefits when administered in adequate amounts. Common genera such as “*Lactobacillus*”, “*Bifidobac-terium*”, “*Bacillus*”, “*Streptococcus*”, and “*Enterococcus*” are found in foods and supplements. To be considered a probiotic, a strain must be well-identified, safe, supported by clinical trials, and maintain viability throughout its shelf life [62]. These strains improve gut microbiota balance, enhance immune function, and may mitigate diseases, including colorectal cancer (CRC).

Probiotics affect CRC through complex mechanisms, including immune modulation, Treg cell expansion, cytokine regulation, and the production of anticancer compounds. They bolster antioxidant defenses, break down carcinogens, inhibit harmful bacteria, and strengthen gut barrier integrity. Probiotics also induce apoptosis in CRC cells, potentially inhibiting tumor growth [3]. Research using animal models has shown that probiotics like “*Faecalibacterium prausnitzii*” and “*Lactobacillus acidophilus*” can prevent CRC through anti-inflammatory pathways and tumor inhibition. Similar effects were observed with other lactic acid bacteria, such as “*Bifidobacterium longum*” and “*Streptococcus thermophilus*” [16]. In CRC patients, probiotics enhanced mucosal barrier integrity and reduced tumor atypia post-surgery [3]. Various single and multistrain probiotics, including VSL#3, have been studied for CRC management. These interventions alter gut microbiota, increasing beneficial bacteria and reducing CRC-associated taxa. Probiotics reduce postoperative complications, improve quality of life, enhance gastrointestinal function, and decrease inflammation [16]. Moreover, probiotics can disrupt biofilms and inhibit biofilm-associated pathogens by competing for resources, modulating immune responses, and producing antibiofilm substances [3]. This underscores their role in managing CRC complications. Specific strains like “*Streptococcus thermophilus*” inhibit CRC tumor growth and promote favorable microbiota composition by reducing non-LAB species [16]. “*Lactobacilli*” and “*Bifidobacteria*” have demonstrated efficacy in reducing tumor progression in models such as AOM/DSS mice. These effects are attributed to enhanced SCFA production, apoptosis promotion, and cell proliferation inhibition. Certain probiotics also alleviate chemotherapy and radiotherapy side effects [63]. Overall, probiotics have significant potential in CRC prevention and treatment, as highlighted in Table 1, which summarizes their anti-carcinogenic effects and mechanisms.

### 8.2. Prebiotics

Prebiotics are compounds like polyunsaturated fatty acids (PUFAs), polyphenols, and specific carbohydrates (inulin, fructooligosaccharides (FOS), galactooligosaccharides (GOS), xylooligosaccharides (XOS)) that selectively ferment to alter the gut microbiota, promoting beneficial bacteria growth while inhibiting pathogens. This results in anti-inflammatory effects and gut health improvements [3,73,74]. Animal studies show that incorporating FOS and GOS modulates gut microbiota, enhancing strains such as “*Lactobacillus*”, “*Bifidobacterium*”, and “*Akkermansia*”, leading to benefits like reduced weight, improved gut barrier integrity, and better glucose tolerance [3,75]. Prebiotics like GOS, inulin, and phenolic compounds have demonstrated CRC inhibition in genetically predisposed or carcinogen-exposed models. FOS has shown beneficial effects on CRC in human colon cell lines, and combinations like GOS with inulin exhibit enhanced preventive activity, reducing colon cancer biomarkers in murine models [3,76,77]. These findings imply that prebiotic interventions, especially in combination, could be valuable in CRC prevention, promoting gut health and reducing cancer risk [78]. Prebiotics’ scope now includes not only non-digestible oligosaccharides but also compounds like conjugated linoleic acid, PUFAs, SCFAs, and dietary polyphenols [62]. Consuming prebiotics is linked to anti-carcinogenic effects, primarily through SCFA production during fermentation. SCFAs exhibit anti-inflammatory actions, stimulate Treg cells, and lower interferon-gamma (IFN-γ) levels, while prebiotics prevent pathogen binding and detoxify genotoxic compounds. These effects often complement probiotics [62]. Table 2 provides an extensive overview of prebiotic sources and their effects on CRC, underscoring their role in dietary strategies for enhancing gut health and lowering cancer risk.

### 8.3. Postbiotics

Postbiotics are bioactive compounds produced during the fermentation of probiotic cells in the gut. Defined by the International Scientific Association of Probiotics and Prebiotics (ISAPP) as non-viable microorganism preparations, postbiotics include inactivated microbial cells and their components [83]. They are considered safer than live microbes and offer health benefits by selectively inhibiting tumor cells, enhancing intestinal health, and modulating immune responses. These properties allow researchers and healthcare providers to explore new methods for improving gut health and potentially preventing colorectal cancer. Postbiotics, such as those from “*Saccharomyces boulardii*”, have demonstrated anti-tumor effects by reducing cell viability, inducing apoptosis, and altering gene expression in colon cancer cells. They also modify gut microbiota composition and boost immune function, aiding colorectal cancer (CRC) treatment while minimizing side effects. The potential of postbiotics as preventive and adjunctive therapies for CRC is notable, offering advantages in economic viability, clinical safety, and stability [84,85]. Table 3 outlines key postbiotic substances and their roles in CRC inhibition, showcasing innovative applications of postbiotics in cancer management and prevention strategies.

### 8.4. Faecal Microbiota Transplantation

Fecal Microbiota Transplantation (FMT) is a biotherapy involving the transfer of fecal material from a healthy donor to a patient to restore a healthy gut microbiome, primarily approved for treating *Clostridium difficile* infection (CDI) [91,92,93]. Beyond CDI, FMT shows promise in addressing obesity, metabolic conditions, and various gastrointestinal disorders by rebalancing microbial communities [94]. Research suggests that restoring microbiomes through FMT may significantly impact overall health. For instance, Rosshart et al. found that fecal transplants from wild mice improved lab mice’s resistance to chemically induced colorectal cancer (CRC) [95]. Conversely, Wong et al. reported that microbiota from CRC patients increased tumor incidence and inflammation in mice, underscoring the potential adverse effects of dysbiotic microbiomes in cancer [96]. These studies highlight the dual role of microbiomes in CRC: promoting health or tumorigenesis, depending on microbial composition. Combining FMT with anti-PD-1 therapy has shown synergistic effects in CRC treatment, supported by metagenomic and metabolomic analyses [97]. This approach underscores the importance of selecting donors and microbiome compositions to optimize therapeutic outcomes. FMT’s integration with cancer immunotherapies could lead to personalized treatments, enhancing treatment responses and patient outcomes in CRC by targeting the microbiome.

### 8.5. Bacteriophage Therapy

Bacteriophage therapy emerges as a promising treatment for colorectal cancer (CRC) through the modulation of gut microbiota. Bacteriophages are viruses that specifically infect bacteria, altering bacterial composition and metabolic processes in the gut. This therapy can modulate the immune system and address disorders associated with dysbiosis, such as obesity and cancer, by targeting harmful bacteria while preserving beneficial ones [98]. Although the mechanisms of phage therapy in cancer are not fully understood, studies with lytic bacteriophage EFA1 and bioinorganic hybrid bacteriophage M13@Ag show promising effects, including biofilm disruption, a reduction in immunosuppressive myeloid-derived suppressor cells (MDSCs), and the potential enhancement of immunotherapy and chemotherapy efficacy [99]. Disrupting biofilms is crucial as they protect pathogenic bacteria and tumor cells, making them resistant to treatment. By reducing MDSCs, phage therapy may enhance anti-tumor immune responses and support existing cancer treatments. These findings suggest integrating phage therapy into multi-modal CRC treatment approaches, potentially augmenting the effectiveness of conventional therapies and modifying the gut microbiome to improve outcomes. While phage therapy shows potential in targeting cancer-related bacteria and optimizing the tumor microenvironment, further research is needed to clarify interactions and any unintended consequences. In summary, bacteriophage therapy is a viable and innovative approach for CRC treatment. Ongoing research is essential for optimizing this therapy, understanding mechanisms, improving safety, and maximizing therapeutic benefits in CRC management.

### 8.6. Fiber Diet

Dietary interventions, especially those incorporating probiotic-rich foods, are promising for colorectal cancer (CRC) prevention [100]. For example, “*Lactobacillus gasseri*” and “*Cudrania tricuspidata*” leaf extracts have shown efficacy in reducing tumor count and inflammatory gene expression. Foods rich in fibers, polysaccharides, and soy-based products impact CRC risk through microbiota-driven mechanisms. Cruciferous vegetables, high in bioactive compounds, reduce CRC risk by converting glucosinolates into sulforaphane, highlighting the link between diet, food processing, and the gut microbiome. Additionally, berries are metabolized by gut microflora into urolithin, which inhibits pathways relevant to CRC prevention. This underscores the intricate interplay between diet, microbiota, and CRC mitigation, emphasizing the need to understand dietary interventions’ influence on the gut environment. Beneficial probiotic strains’ anti-tumor effects in preclinical models further stress the importance of dietary strategies in promoting gut health and reducing cancer risk [101,102].

## 9. Gut Microbiota Impact on Gastrointestinal Therapy

The gut microbiota significantly influences the success of anti-cancer treatments, with the microbiome playing a crucial role in modulating immune responses [20]. Optimizing the microbiome has the potential to enhance therapeutic efficacy and reduce side effects, leading to more effective and tolerable gastrointestinal cancer treatments. As research progresses, it offers exciting possibilities for personalized medicine, where microbiota composition is a key factor in tailoring treatments [20]. There is significant proof backing the utilization of probiotics, prebiotics, and postbiotics in the management of CRC. Probiotics, including lactic acid bacteria and *Bifidobacterium*, can influence gut microbiota, decrease inflammation, and boost the immune response, potentially enhancing the effectiveness of chemotherapy and minimizing side effects [103]. Prebiotics such as fructooligosaccharides (FOS), galactooligosaccharides (GOS), and inulin enhance the proliferation of helpful gut bacteria, altering gut microbiota structure and function and possibly lowering the risk of CRC [104]. Postbiotics, consisting of non-living bacterial products or their metabolic byproducts, can influence the immune system, reduce cancer cell growth, and uphold the integrity of the intestinal barrier, demonstrating potential in CRC treatment and possibly exhibiting anti-tumor properties [105]. Cancer treatment outcomes, including immunotherapy and chemotherapy, are closely linked to gut microbiota composition. “*Bifidobacterium breve*” enhances anti-PD-1 immunotherapy, while the success of CTLA-4 blockade relies on “*Bacteroides*” species. Platinum-based chemotherapy efficacy is also dependent on healthy gut microbiota. Responders to anti-PD-1 therapy often have a higher abundance of “*Faecalibacterium*”, whereas non-responders typically show more “*Bacteroidales*” species [106]. Research on germ-free, antibiotic-treated, or microbiota-deficient mice reveals reduced effectiveness of chemotherapy agents. Specifically, “*Fusobacterium nucleatum*” has been associated with negative outcomes, such as lower relapse-free survival and poor responses to 5-Fluorouracil (5-FU), as it inhibits apoptosis in CRC models [107]. These findings emphasize the detrimental impact of certain gut bacteria, highlighting the need for microbiome-targeted interventions to enhance treatment efficacy. Treatments are significantly impacted by differences in the gut microbiota between CRC subtypes, such as microsatellite instability-high (MSI-H) and microsatellite stable (MSS). *Fusobacterium nucleatum* and other bacteria that can stimulate inflammation and carcinogenesis are linked to MSI-H tumors, which frequently show greater microbial diversity [108]. On the other hand, bacteria like *Bacteroides fragilis* and *Escherichia coli*, which can create toxins that damage DNA and encourage the formation of cancer, are associated with MSS tumors, which often have decreased microbial diversity [109]. Due to the increased mutational burden and particular bacterial interactions, MSI-H CRC patients typically respond better to immune checkpoint inhibitors, which is one way that these distinctions impact therapeutic responses [110]. The gut microbiome impacts chemotherapeutic efficacy by directly modifying drugs or indirectly affecting cytotoxic mechanisms. Studies show that fecal microbiota transplantation from responders enhances ICI therapy sensitivity in non-responders. Key bacteria positively impacting ICI responses include “*Akkermansia* spp.”, “*Bifidobacterium* spp.”, “*Roseburia* spp.”, and “*Faecalibacterium* spp.”, although their effects can vary across cohorts. In murine models, certain bacteria like “*Enterococcus* spp.” enhance ICI therapy by activating immune cell infiltration into tumors [91]. Metabolites from “*Lactobacillus delbrueckii*” subsp. “*bulgaricus*”, such as exopolysaccharides, also improve treatment efficacy. Conversely, some “*E. coli*” strains can reduce cytotoxic T cells during anti-PD-1 treatment. This research underscores the pivotal role that specific bacteria and metabolites play in shaping treatment responses in colorectal cancer, suggesting that microbiome-based strategies could optimize therapeutic outcomes for patients undergoing ICI therapy [111]. Specific bacterial species like *Bacteroides fragilis* and *Akkermansia muciniphila* have been demonstrated to improve the effectiveness of ICIs by stimulating T-cell activation and boosting antitumor immunity [112]. In contrast, organisms such as *Bacteroides thetaiotaomicron* and *Escherichia coli* may reduce the efficacy of ICIs by creating immunosuppressive conditions [113]. Table 4 emphasizes the roles of gut microbiota in health and disease, including their influence on cancer therapies, and the use of AI tools to analyze microbiome data for disease prediction and treatment.

## 10. Conclusions

Colorectal cancer (CRC) is a major health concern due to its high prevalence and mortality rate. Understanding its causes is crucial, with the intestinal microbiome being a significant factor in human physiology, affecting metabolic and immunological processes. Disruptions in gut homeostasis and microbiota composition may contribute to CRC development. Identifying microbial profiles associated with CRC can serve as valuable biomarkers for the disease’s early detection and prognosis. Moreover, therapeutic strategies targeting microbiota modulation present promising avenues for CRC prevention and treatment. To ensure the accuracy of microbiota research, study cohorts must be meticulously planned, considering factors like geography, age, diet, BMI, medications, antibiotic use, and pet ownership. Additionally, maintaining microbiota integrity and preventing contamination during sample collection is essential.

## 11. Prospects

**Figure 4:**
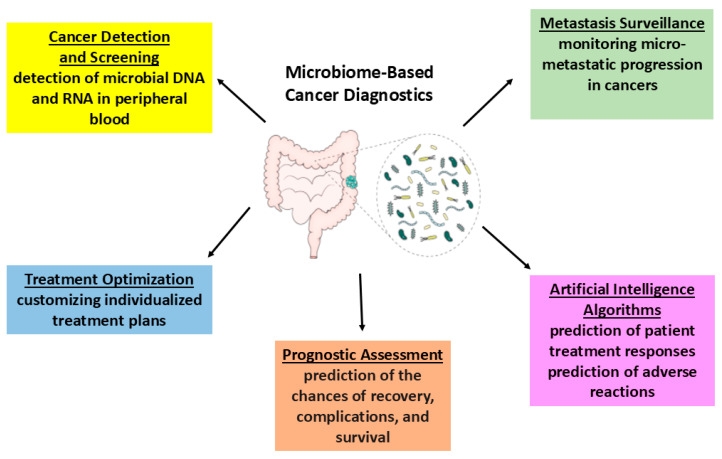
Potential of microbiome analysis-derived data to advance novel diagnostic capabilities in cancer.

Future trials should emphasize personalized and integrated approaches, considering individuals’ clinical and pathological histories to optimize microbiota-targeted treatments while minimizing side effects. Advancements in leveraging the gut microbiome for CRC prevention and treatment hold the potential to significantly improve patient outcomes. Recognizing dysbiosis indicators may elucidate disease progression and therapeutic variability in patients with similar clinical profiles. Interventions like dietary changes, prebiotics, probiotics, symbiotics, postbiotics, fecal microbiota transplantation, and bacteriophage therapy aim to restore gut microbiome balance and health. Dysbiosis is linked to various diseases, particularly gastrointestinal cancers, and microbiota interventions could serve in cancer prevention and treatment. However, careful monitoring and consideration of the benefit-to-risk ratio are essential. Emerging scientific evidence opens new avenues in cancer diagnosis and management, with disease-associated microorganisms potentially serving as biomarkers to enhance personalized medicine strategies. Continued research is crucial for translating these insights into effective cancer care interventions. In the meantime, dietary interventions, such as increased fiber intake, bioactive substance enrichment, healthy nutritional practices, and prudent antibiotic use are recommended to prevent dysbiosis-related health conditions, including GI cancers. Examining intratumoral microbiota as diagnostic and prognostic tools marks a significant advancement in cancer treatment strategies. Integrating microbial profiling into clinical practice can improve diagnostic accuracy and the personalization of treatments. Microbiome analysis data may advance novel diagnostic capabilities, including microbial DNA and RNA detection in peripheral blood, monitoring micro-metastatic progression, evaluating prognosis, customizing treatment plans, and using artificial intelligence for predictive analytics (Figure 4). Developing microbiota-focused strategies, particularly in the prevention and treatment of GI cancers, presents promising opportunities for improving health outcomes. As research progresses, these strategies could greatly enhance cancer management and provide new therapeutic pathways.

## Figures and Tables

**Table 1:** Bacterial species with anti-carcinogenic effects: The table lists bacterial species known for their anti-carcinogenic properties and their suggested mechanisms of action, such as enhancing immune responses, producing short-chain fatty acids (SCFAs), and modulating inflammation. Relevant studies supporting these effects are also included.

General	Species	Effect	Mechanism	Reference
*Lactobacillus*	*L. acidophilus*	Anti-inflammatory activity	Modulation of *MAPK* Signaling Pathway and *TLR-2* Mediated *NF-κB* Activation: Can modulate the *MAPK* signaling pathway and *TLR-2*-mediated *NF-κB* activation in inflammatory intestinal epithelial cells (IECs), thereby regulating inflammatory responses. Reduction in IL-8 Secretion and Inflammatory Marker Expression: Reduce the secretion of interleukin-8 (IL-8) and the expression of phosphorylated *NF-κB* (p-p65), p-p38 *MAPK*, *VCAM-1*, and *COX-2* in inflammatory conditions, indicating decreased inflammation. Enhanced TLR-2 Expression in LPS- and TNF-α-Induced Inflammatory IECs: Combining specific probiotics, such as *Bifidobacterium lactis*, enhances TLR-2 expression in intestinal epithelial cells subjected to LPS and TNF-α, improving immune response against inflammation.	[64]
	*L. plantarum* (*L. plantarum* L125)	Anti-proliferative, anti-clonogenic, and anti-migration activity using cell-free culture supernatant	Anti-Proliferative Effect Mediated by Cell Surface Molecules and Excreted Metabolites: Exhibit anti-proliferative effects through cell surface molecules and excreted metabolites, including exopolysaccharides (EPS), peptidoglycans, conjugated linolenic acids (CLA), and S-layer proteins, which have been implicated in inducing cell death.	[65]
	*L. rhamnosus*	Arrest cancer cell growth Apoptosis induction Synergistic action with 5-Fluorouracil and Irinotecan Anti-proliferative activity	Secretion of Bioproducts by Lactobacillus rhamnosus GG (LGG): Secretes bioproducts that trigger a reduction in cancer cell viability. Induction of Mitotic Arrest: Induces mitotic arrest in the G2/M phase of the cell cycle, preventing cell division. Synergistic Action with Chemotherapy: Exhibits a positive synergistic effect with both 5-Fluorouracil and Irinotecan by sensitizing cancer cells and increasing pro-apoptotic gene expression. Activation of Intrinsic Mitochondrial Pathway: The activation of Bax leads to the intrinsic mitochondrial pathway, promoting apoptosis. Apoptosis Induction: Apoptosis is induced via the activation of caspase-3 and caspase-9, alongside the release of cytochrome c. Decrease in Anti-Apoptotic and Proliferative Genes: Results in a decrease in the expression of anti-apoptotic genes, such as *Bcl-2*, and genes involved in cell cycle progression, including *cyclin D1* and *cyclin E*, as well as *ERBB2*, which is associated with cell proliferation.	[66,67]
	*L. casei ATCC393*	Cell viability reduction Apoptosis induction	Modulation of Genes Involved in Cell Cycle Progression and Tumor Growth Probiotic treatment modulates the expression of genes associated with cell cycle progression and tumor growth. Downregulation: Survivin (*BIRC5*): the downregulation of survivin expression reduces anti-apoptotic activity, thereby promoting apoptosis in cancer cells. *CCND1*: Downregulation of *CCND1*, which encodes cyclin D1, is crucial for cell growth and progression. Upregulation: Selective Induction of Apoptosis: Upregulation of certain pathways facilitates selective apoptosis in various tumor cell lines while sparing normal cells and tissues, primarily through the activation of intrinsic apoptosis signaling pathways.	[68]
*Bifidobacterium*	*B. lactis*	Apoptosis induction	Induction of Apoptosis Pathways: The combination of specific *Bifidobacterium* species induces both intrinsic and extrinsic apoptosis pathways, enhancing the apoptotic response in tumor cells. Inhibition of *NF-κB* Activity: The presence of certain *Bifidobacterium* species leads to the inhibition of Nuclear Factor kappa-light-chain-enhancer of activated B cells (*NF-κB*) activity, which is crucial for regulating inflammatory responses and tumor survival.	[69]
	*B. longum*	Anti-inflammatory and cancer prevention effects	Suppression of IκB-α Degradation: By suppressing the degradation of *IκB-α*, there is a subsequent decline in its translocation into the nucleus and its DNA binding activity. This results in decreased concentrations of NF-κB and interleukin-6 (IL-6), thereby reducing inflammation and tumor progression. Inhibition of Proliferation and Metastasis: The inhibition of cyclooxygenase-2 (COX-2), an NF-κB-dependent mediator, leads to the suppression of proliferation and metastasis in colitis-related cancer, thus contributing to a decrease in oncogenic signaling pathways.	[70]
*Streptococcus*	*S. thermophilus*	Anti-proliferative protective. CRC cell viability reduction	Production of β-Galactosidases: The production of β-galactosidases inhibits the Warburg metabolic phenotype and disrupts the Hippo signaling pathway in colorectal cancer (CRC) cells, leading to decreased tumor cell proliferation and survival. Increased Abundance of Beneficial Probiotic Species: The treatment increases the abundance of beneficial probiotic species, such as *Bifidobacterium* and *Lactobacillus*, while simultaneously reducing pathogenic bacteria associated with CRC, thereby promoting a healthier gut microbiota composition.	[71]
*Faecalibacterium*	*F. prausnitsii*	Anti-inflammatory effect	Production of Microbial Anti-inflammatory Molecule (MAM) The probiotic produces a protein known as the Microbial Anti-inflammatory Molecule (MAM), which exerts inhibitory effects on the *NF-κB* pathway in various intestinal epithelial cells. This action results in a reduction in colitis in mouse models, highlighting its potential in mitigating intestinal inflammation.	[72]

**Table 2:** Prebiotic substances, sources, and their major effects on colorectal cancer (CRC): the table summarizes various prebiotic substances, their primary food sources, and the significant effects they have on CRC prevention and management. Key associations between prebiotics and the modulation of the gut microbiota, the enhancement of immune response, and the reduction in inflammation are highlighted.

Prebiotic Substance	Source	Effect	Reference
Polyunsaturated fatty acids (PUFAs)	Abundant in fish and fish oil supplements	Modulation of Eicosanoid Profiles by ω-3 PUFA Omega-3 polyunsaturated fatty acids (PUFAs) modulate eicosanoid profiles in both circulation and colorectal tumor tissues. This modulation leads to a reduction in ω-6-series metabolites while increasing the levels of ω-3-series metabolites, such as docosahexaenoic acid (DHA, 22:6ω-3) and eicosapentaenoic acid (EPA, 20:5ω-3), which play significant roles in mediating anti-cancer and anti-angiogenic effects. Antineoplastic Effects of EPA Eicosapentaenoic acid (EPA) is thought to exert its antineoplastic effects by inhibiting cyclooxygenase-2 (COX-2)-dependent synthesis of prostaglandin E2 (PGE2) and reducing the de novo production of PGE3, thereby contributing to the suppression of tumor growth.	[79,80]
Polyphenols	Jaboticaba (*Myrciaria jaboticaba*) seed extract (LJE)	Pro-Oxidant and Cytotoxic Effects of Phenolic Compounds Phenolic compounds, including castalagin, vescalagin, procyanidin A2, and the ellagic acid found in LJE, exhibit pro-oxidant and cytotoxic effects against cancer cells, contributing to the inhibition of tumor cell proliferation. Antioxidant Properties These phenolic compounds also demonstrate antioxidant properties by reducing the generation of reactive oxygen species (ROS) in normal human cell lines, such as IMR90, thereby protecting healthy cells from oxidative stress.	[81]
Inulin	Garlic, onion, artichoke, asparagus, and chicory	Increase in Propionate-Producing Bacteria There is an increase in well-known propionate-producing bacterial species from the *Bacteroidaceae* and *Prevotellaceae* families, which are beneficial for gut health and linked to anti-inflammatory effects. Reduction in Firmicutes and ***Ruminococcus gnavus*** A reduction in *Firmicutes*, particularly *Ruminococcus gnavus*, has been observed, which corresponds to a decrease in inflammatory conditions associated with gut dysbiosis. Increase in the ***Faecalibacterium*** genus An increase in the *Faecalibacterium* genus, recognized for its butyrate-producing capabilities, is noted. This genus exhibits anti-inflammatory effects primarily through the inhibition of NF-κB signaling, contributing to improved gut homeostasis.	[82]

**Table 3:** Examples of postbiotic substances and their suggested roles in colorectal cancer (CRC) inhibition: examples of postbiotic substances, along with their proposed mechanisms of action in inhibiting colorectal cancer (CRC). It outlines how these bioactive compounds, derived from the fermentation of probiotics, contribute to cancer prevention through mechanisms such as the modulation of inflammation, the enhancement of apoptosis, and the support of gut barrier integrity.

Substance	Example	Source	Effect	Reference
Inactivated microbial cells	Heat-killed LGG	*Lactobacillus rhamnosus* *GG*	Increase in Anti-Inflammatory Mediators Substances lead to an increase in anti-inflammatory mediators, promoting a more balanced immune response and reducing overall inflammation. Decreased Inflammatory Response to *E. coli* Lipopolysaccharides (LPS) These substances also decrease the inflammatory response induced by *E. coli* lipopolysaccharides (LPS), thereby mitigating the effects of pathogenic stimuli and contributing to intestinal health.	[86]
Cell components	Amuc_1100	A protein from the outer membrane of *Akkermansia muciniphila*	Induction of Anti-Tumor Immune Response These substances induce an anti-tumor immune response, enhancing the body’s ability to combat cancer cell growth. Improvement of Colitis through CTL Modulation They also improve colitis by modulating cytotoxic T lymphocyte (CTL) activity, resulting in increased CTL concentrations in both the colon and mesenteric lymph nodes. This modulation upregulates CTL activity and tumor necrosis factor-alpha (*TNF-α*), leading to the apoptosis of tumor cells.	[87]
	Lipoteichoic acid (LTA)	Cell wall component of *Lactobacillus paracasei* D3-5	Reduction in Age-Related Leaky Gut and Inflammation These substances reduce age-related leaky gut and associated inflammation by modulating the *TLR-2/p38*-*MAPK/NF-κB* signaling pathway, leading to the increased expression of mucin (*Muc2*), which enhances the intestinal barrier function.	[88]
Metabolites secreted by gut microbiota.	SCFAs including acetate, propionate and butyrate; enzymes; bacteriocins; reuterin; acetoin; organic acids	Members of gut microbiota example: *Faecalibacterium prausnitzii*	Inhibition of CRC Cell Migration and Invasion by Butyrate Butyrate blocks the motility-dependent activation of histone deacetylase 3 (HDAC3)-dependent signaling pathways, resulting in the inhibition of migration and the invasion of colorectal cancer (CRC) cells. Antitumor Immune Response Triggered by Butyrate and Propionate Both butyrate and propionate trigger an antitumor immune response by activating a positive feedback loop that upregulates genes involved in immune activation. This process promotes the activation of cytotoxic T lymphocytes (CD8+) and enhances the secretion of interferon-gamma (IFN-γ).	[89,90]
Cell-free supernatants (CFS)	Exopolysaccharides (EPS), peptidoglycans and conjugated linolenic acids (CLA)	*L. plantarum* (*L. plantarum* L125)	Induction of Cell Death through Surface Molecules and Metabolites Cell death is induced through interactions with cell surface molecules and/or the action of excreted metabolites, including exopolysaccharides (EPS), peptidoglycans, conjugated linolenic acids (CLA), and S-layer proteins, which collectively promote apoptotic pathways in target cells.	[65]

**Table 4:** Microbial Impacts, Therapeutic Targets, and AI Applications in Microbiome Research.

Category	Details	Effect	Reference
Microbial Impacts	Short-Chain Fatty Acids (SCFAs)	Produced by fermentation of dietary fibers; anti-inflammatory and anti-carcinogenic properties.	[55]
	Secondary Bile Acids	Produced by gut bacteria from primary bile acids; can induce DNA damage and promote carcinogenesis.	[114]
Therapeutic Targets	*Bacteroides fragilis*	Enhances efficacy of immune checkpoint inhibitors (ICIs) by promoting T-cell activation.	[115]
	*Akkermansia muciniphila*	Associated with increased infiltration of CD8+ T cells into tumors.	[116]
	*Bacteroides thetaiotaomicron*	Can inhibit effectiveness of ICIs by inducing immunosuppressive environments.	[116]
AI Applications	DeepMicro	Utilizes high-throughput sequencing data to detect patterns, classify microbes, and predict disease associations.	[117]
	Meta-Spec	Integrates host and microbial information to map microbiome patterns related to diseases.	[118]
	MicroPheno	Predicts phenotypic traits from microbiome data, aiding in the identification of disease-associated microbial features.	[118]

## Data Availability

The original contributions presented in this study are included in the article; further inquiries can be directed to the corresponding author.
